# Assessing the agreement between a global navigation satellite system and an optical-tracking system for measuring total, high-speed running, and sprint distances in official soccer matches

**DOI:** 10.1177/00368504231187501

**Published:** 2023-07-10

**Authors:** Piotr Makar, Ana Filipa Silva, Rafael Oliveira, Marcin Janusiak, Przemysław Parus, Małgorzata Smoter, Filipe Manuel Clemente

**Affiliations:** 1 Gdańsk University of Physical Education and Sport, Gdańsk, Poland; 2 Escola Superior Desporto e Lazer, 112031 Instituto Politécnico de Viana do Castelo, Viana do Castelo, Portugal; 3 Research Center in Sports Performance, Recreation, Innovation and Technology (SPRINT), Melgaço, Portugal; 4 The Research Centre in Sports Sciences, Health Sciences and Human Development (CIDESD), Vila Real, Portugal; 5 Sports Science School of Rio Maior–Polytechnic Institute of Santarém, Rio Maior, Portugal; 6 Life Quality Research Centre, Rio Maior, Portugal; 7 Śląsk Wrocław Basketball, Physiology Department, Wrocław, Poland; 8 FC WKS Śląsk Wrocław, Physical Performance Department, Wrocław, Poland; 9 Department of Basics of Physiotherapy, Gdansk University of Physical Education and Sport, Gdańsk, Poland; 10 Instituto de Telecomunicações, Delegação da Covilhã, Lisboa, Portugal

**Keywords:** **:** Football, athletic performance, player tracking systems, training load monitoring, locomotor demands

## Abstract

This study aimed to compare the agreement of total distance (TD), high-speed running (HSR) distance, and sprint distance during 16 official soccer matches between a global navigation satellite system (GNSS) and an optical-tracking system. A total of 24 male soccer players, who are actively participating in the Polish Ekstraklasa professional league, were included in the analysis conducted during official competitions. The players were systematically monitored using Catapult GNSS (10-Hz, S7) and Tracab optical-tracking system (25-Hz, ChyronHego). TD, HSR distance, sprint distance, HSR count (HSRC), and sprint count (SC) were collected. The data were extracted in 5-min epochs. A statistical approach was employed to visually examine the relationship between the systems based on the same measure. Additionally, *R*^2^ was utilized as a metric to quantify the proportion of variance accounted for by a variable. To assess agreement, Bland–Altman plots were visually inspected. The data from both systems were compared using the estimates derived from the intraclass correlation (ICC) test and Pearson product–moment correlation. Finally, a paired *t*-test was employed to compare the measurements obtained from both systems. The interaction between Catapult and Tracab systems revealed an *R*^2^ of 0.717 for TD, 0.512 for HSR distance, 0.647 for sprint distance, 0.349 for HSRC, and 0.261 for SC. The ICC values for absolute agreement between the systems were excellent for TD (ICC = 0.974) and good for HSR distance (ICC = 0.766), sprint distance (ICC = 0.822). The ICC values were not good for HSRCs (ICC = 0.659) and SCs (ICC = 0.640). *t*-test revealed significant differences between Catapult and Tracab for TD (*p* < 0.001; *d* = −0.084), HSR distance (*p* < 0.001; *d* = −0.481), sprint distance (*p* < 0.001; *d* = −0.513), HSRC (*p* < 0.001; *d* = −0.558), and SC (*p* < 0.001; *d* = −0.334). Although both systems present acceptable agreement in TD, they may not be perfectly interchangeable, which sports scientists and coaches must consider when using them.

## Introduction

Monitoring locomotor demands through technological devices has become a widespread and recurring practice in soccer training. A survey conducted among 94 coaches and 88 practitioners belonging to elite English soccer found that tracking systems (e.g., global navigation satellite system [GNSS]) were used more than other different training load monitoring methods (e.g., blood lactates, ratings of perceived exertion, heart rates).^1^ In another survey conducted among 82 high-level soccer teams competing in the top leagues of countries such as the United Kingdom, Spain, France, Germany, and Italy, the findings revealed that approximately 40% of the teams utilized time motion analysis and accelerometers as the primary tools for quantifying training load.^2^ It is noteworthy to acknowledge that the term “training load” has been a topic of discussion regarding its accuracy. This is primarily due to the conventional association of the term “load” with a mechanical variable measured in newtons within the International System of Units.^3^ However, it is crucial to emphasize that in the specific context of training load, it serves as a scientific construct rather than a direct “measurement” per se.^4^ Therefore, its usage does not contravene any scientific principles.^4^

To enhance the efficacy of collecting sports science training data, it is crucial to acknowledge the inherent value of coaches and performance staff. This recognition plays a pivotal role in facilitating the widespread adoption and seamless integration of meticulously engineered tracking systems that are purposefully designed to augment training load practices. Supporting this notion, a comprehensive survey was conducted among 176 soccer coaches and performance coaches, revealing that sport science data holds substantial importance in guiding their practice, being perceived as both somewhat important and very important.^5^

In light of the proliferation of tracking systems, it is evident that a multitude of options are now available on the market. However, it becomes increasingly challenging to conduct fair comparisons and establish the interchangeability of data among these diverse alternatives.^6^ Among the various technological options, GNSS, ultra-wideband technology, and optical video tracking systems are prominent examples. The usability of these options is contingent upon specific contexts and objectives. GNSS, while cost-effective, is primarily applicable in outdoor facilities. Conversely, ultra-wideband technology, despite its higher cost, offers versatility by functioning effectively in both outdoor and indoor settings.^7^

In the context of outdoor facilities, GNSS remains widely utilized, potentially owing to its user-friendly nature compared to installation-dependent optical video tracking systems, as well as its relatively lower cost in comparison to ultra-wideband technology. It is important to note, however, that optical video tracking systems can present an alternative and intriguing solution for sports scientists and players in outdoor facilities. These systems ensure high-quality data collection and provide the opportunity to combine time-motion analysis with notational analysis, all without necessitating any devices to be placed on the players. Having in mind the multiplicity of the options and technical aspects of the devices, it is particularly essential to focus on the agreement between such systems,^7^ considering that data collected can vary from system to system with a remarkable impact on data interpretation.

Taking that into consideration, different studies have focused on testing absolute agreement between different tracking systems.^6,8,9^ For example, a 10-Hz multi-GNSS GNSS device (vector, Catapult) and two optical tracking systems (25-Hz Tracab and Second Spectrum) were compared. The results indicated that in comparison to GNSS, Tracab revealed significantly higher values for most locomotor measures followed by the other optical system (Second Spectrum).^6^ Another study comparing two 10-Hz GNSS systems (Viper, StatSports; and Apex, StatSports) with Tracab optical tracking system demonstrated significant differences between GNSS and the optical tracking systems for locomotor measures such as total distance (TD), high-speed running (HSR) distance, and sprint distance.^8^ However, the different systems presented very large correlations.^8^ Another comparison of Tracab and a 10-Hz GNSS (Wimu) revealed that the optical tracking system slightly overestimated most locomotor measures compared to GNSS.^9^

These aforementioned studies^6,8,9^ suggest notable differences in locomotor measures when comparing different technologies, specifically GNSS and optical video tracking systems. However, they also demonstrate a strong correlation between the two, indicating the potential for interchangeability. Despite these findings, the current research has not focused on analyzing peak demands within 5-min epoch periods. Peak demands, or worst-case scenarios, have recently garnered attention,^10^ raising concerns about the accuracy, precision, and interchangeability of different systems.

Although Ellens’ study^6^ investigated the interchangeability between a 10-Hz GNSS Vector and Tracab (an optical tracking system with 25-Hz), the analysis did not specifically examine the interchangeability of epoch periods. Therefore, further research is needed to determine whether interchangeability can also be achieved within 5-min epochs. Such research would provide another independent assessment of absolute agreement between different systems and models, particularly comparing new GNSS systems like the Catapult S7 with Tracab (an optical tracking system with 25-Hz).

Testing for interchangeability holds significant importance for several reasons. Firstly, it allows for better control over the comparisons made between scientific articles and benchmarks conducted on players. By establishing the interchangeability of devices and technologies, it becomes possible to ensure the validity and reliability of such comparisons.

Secondly, considering that clubs often change their devices and technologies, having access to interchangeability values becomes crucial. It provides clubs with the necessary information to determine whether fair and accurate comparisons can be made or if caution should be exercised due to potential discrepancies between different measurement systems. This knowledge empowers clubs to make informed decisions regarding the compatibility and comparability of data collected from different sources.

Therefore, the objective of this study is to assess the absolute agreement between the 10-Hz Catapult S7 GNSS and the 25-Hz Tracab optical video tracking system in terms of TD, HSR distance, sprint distance, as well as the number of HSR and sprints. These measures were specifically chosen due to their significance in quantifying training load within the given context.

TD serves as a comprehensive measure of locomotor demand, which is closely associated with internal load responses.^11^ It provides valuable insights into the magnitude of demands imposed on players. HSR and sprint distances were selected as they represent the most demanding locomotor demands observed during matches. Moreover, these measures are known to have traditionally lower values of precision,^12^ necessitating further examination to ensure optimal accuracy and precision of the collected data.

## Methods

### Study design

This study employed a longitudinal design, focusing on a group of twenty-one soccer players from a single professional team. Over a period of 16 official soccer matches, which took place outdoors in stadium facilities, the players were consecutively observed. The observation period spanned from July 15, 2022, to November 13, 2022, corresponding to the competitive phase of the season. For the analysis, only data from official matches in the domestic competition, including league matches and cup matches, were considered. The players were monitored using two tracking systems: (a) a GNSS and (b) an optical-tracking system. The study aimed to test the agreement between both systems for monitoring locomotor demands of the players during the match.

### Participants

We used nonprobabilistic convenience sampling. A group of 21 male professional football players (231 observations) from the first team of one of the Polish Ekstraklasa clubs (age: 25 ± 3 years, body height: 179.6 ± 5.5 cm, body mass: 76.1 ± 5.0 kg) participated in the research. The data were collected over 16 matches played in the autumn round of the 2022/2023 season and were recorded simultaneously during each of the observed games. Goalkeepers were not included in this study due to the unique nature of their position. Considering the specific movements and actions performed by goalkeepers during matches, the use of GNSS instruments may potentially cause damage or interfere with their typical movements. Hence, to ensure the integrity of the study and avoid any potential harm to the goalkeepers, their data was not collected or analyzed as part of this research. All data were created as a condition of employment where players were routinely monitored throughout league play.

In order to uphold ethical standards, all players involved in the study were provided with detailed information about the study design, the associated risks, and the potential benefits of participation. Only after obtaining their informed consent was the study conducted. The informed consent process ensured that the players were fully aware of the study's objectives, procedures, and potential implications before agreeing to participate. This study adhered to the ethical guidelines outlined in the Declaration of Helsinki for research involving human participants. Confidentiality was strictly maintained throughout the study by anonymizing all data prior to analysis.

### Methodological procedures

Two tracking systems were used simultaneously: (a) a GNSS unit (Vector S7, Catapult Innovations, Melbourne, Australia; 81mm×43mm×16mm), operating at a frequency of 10-Hz and (b) an optical tracking system (TRACAB, ChyronHego, New York, USA) using two multicamera units (each containing three HD-SDI cameras with a resolution of 1920 × 1080 pixels) with a sampling frequency of 25-Hz. On average, the number of satellites connected during data collection was 15, and the average horizontal dilution of precision (HDOP) was 0.7. Vector S7 was preliminarily tested for its ability to assess a force-velocity profile.^13^ Furthermore, the Tracab system underwent a validation process to assess its accuracy and precision in measuring locomotor demands across various running speed thresholds.^14^

The players always used the same GNSS unit to reduce inter-unit variability.^15^ The GNSS units were placed between the players’ shoulder blades and were activated according to a manufacturer's guidelines before kickoff. To avoid potential unit differences, the players wore the same GNSS unit for each match.^8^ The data recorded by the units were downloaded after each match for further analysis using Catapult OpenField Cloud Analytics (OpenField 3.9.0 Catapult Sports, Melbourne, Australia). The following variables were selected for analysis during this study: field time, defined as the time spent on the field (FT; min), TD (m), distance in HSR, defined as a running speed between 19.81 and 25.2 km/h (HSR; m), sprint, defined as velocity greater than 25.2 km/h (SPR; m), High-speed running count (HSRC) and sprint count (SC). The HSR speed threshold of 19.81 km/h was determined based on the research conducted by Abt and Lovell,^16^ who identified this value as the reference for the second ventilatory threshold. This specific threshold has gained broad acceptance and is widely used as a prevalent measure to define arbitrary speed thresholds in soccer players. The selection of the 25.2 km/h speed threshold for sprints aligns with established conventions based on previous research conducted on sprinting in soccer players.^17^ The velocity thresholds chosen are those defined by both tracking system providers. All data from the Tracab system were provided by ChyronHego as a match report. Data from both the Catapult and Tracab systems were extracted in 5-min epochs, which involved dividing the official match time into consecutive 5-min time periods. This approach ensured that all 5-min epochs within the match time were considered and included in the analysis.

### Statistical procedures

Descriptive statistics are presented in the form of mean and standard deviation. Plotting data was performed to visually inspect the relationship between the systems for the same measure. At the same time, *R*^2^ was used as a measure to represent the proportion of the variance for a variable. Measuring agreement was visually inspected by Bland–Altman plots with a 95% confidence interval using the mean difference between measures. The estimates of the intraclass correlation (ICC) test and their 95% confidence intervals were calculated by means of SPSS statistical package (28.0.0.0, IBM, Chicago, IL) based on a mean-rating (k = 2), absolute-agreement, a two-way mixed effects model. The classification of the agreement^18^ was considered good between ICC = 0.75 and ICC = 0.90, while the values above ICC = 0.90 were considered excellent. The Pearson–product correlation test was executed on SPSS (version 28.0.0., IBM, Chicago, USA) for a *p*-value less than 0.05 to analyze the strength of the relationship between the systems. The correlation coefficients^19^ between *r* = 0.50 and *r* = 0.7 were considered large, between *r = *0.7 and *r* = 0.9 very large, and above *r* = 0.9 nearly perfect. The paired *t*-test was used to compare the measures obtained for both systems, followed by the standardized effect size (Cohen's *d*) that was interpreted as^20^: 0.0–0.2, trivial; 0.2–0.5, medium; 0.5–0.8, large; and >0.8, very large. The statistical procedures were executed in SPSS (version 28.0.0., IBM, Chicago, USA) for a *p* < 0.05.

## Results

**Figure 1.**
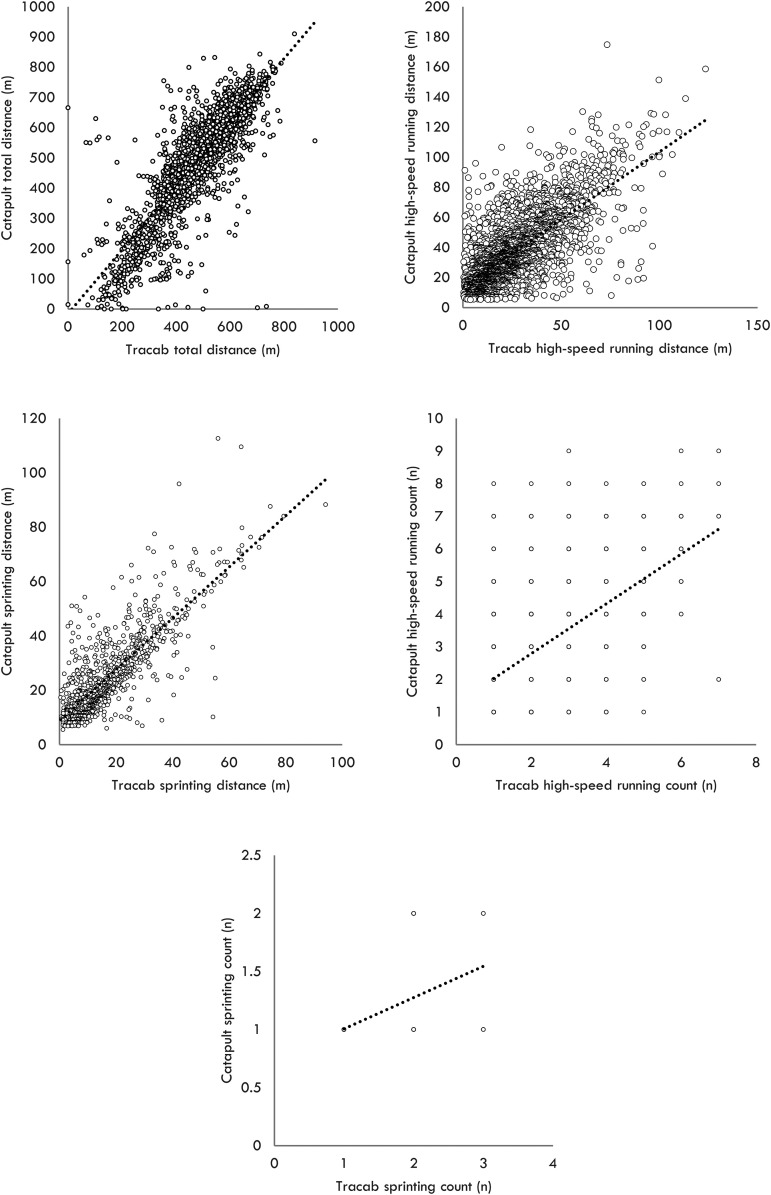
Scatter plot between Catapult and Tracab for TD, HSR distance, sprint distance, HSRC, and SC. The data displayed in the figure represents the aggregation of values over 5-min epochs.

 Figure 1 displays a scatter plot comparing the Catapult and Tracab systems for the various running-based measures extracted during the matches. It is important to note that all data presented in the results correspond to the values obtained for the 5-min epochs. The interaction between Catapult and Tracab systems revealed an *R*^2^ of 0.717 for TD, 0.512 for HSR distance, 0.647 for sprint distance, 0.349 for HSRC, and 0.261 for SC.

 Table 1 presents the ICC and the Pearson–product correlation tests comparing both systems for the different running-based measures. The ICC values for absolute agreement between the systems were excellent for TD (ICC = 0.974) and good for HSR distance (ICC = 0.766), and sprint distance (ICC = 0.822). The ICC values were not good for HSRCs (ICC = 0.659) and SCs (ICC = 0.640).

**Table 1.** ICC and Pearson-product correlation tests comparing both systems for the different running-based measures.

	Intraclass correlation	*r* Pearson
Total distance (m)	0.906 [95% CI: 0.897; 0.914]	0.851 [0.841; 0.860] | *p* < 0.001
High-speed running (m)	0.766 [95% CI: 0.522; 0.864]	0.716 [0.697; 0.734] | *p* < 0.001
Sprint (m)	0.822 [95% CI: 0.445; 0.917]	0.804 [0.780; 0.826] | *p* < 0.001
High-speed running (*n*)	0.659 [95% CI: 0.399; 0.784]	0.590 [0.564; 0.615] | *p* < 0.001
Sprint (*n*)	0.640 [95% CI: 0.542; 0.712]	0.523 [0.468; 0.574] | *p* < 0.001

**Figure 2.**
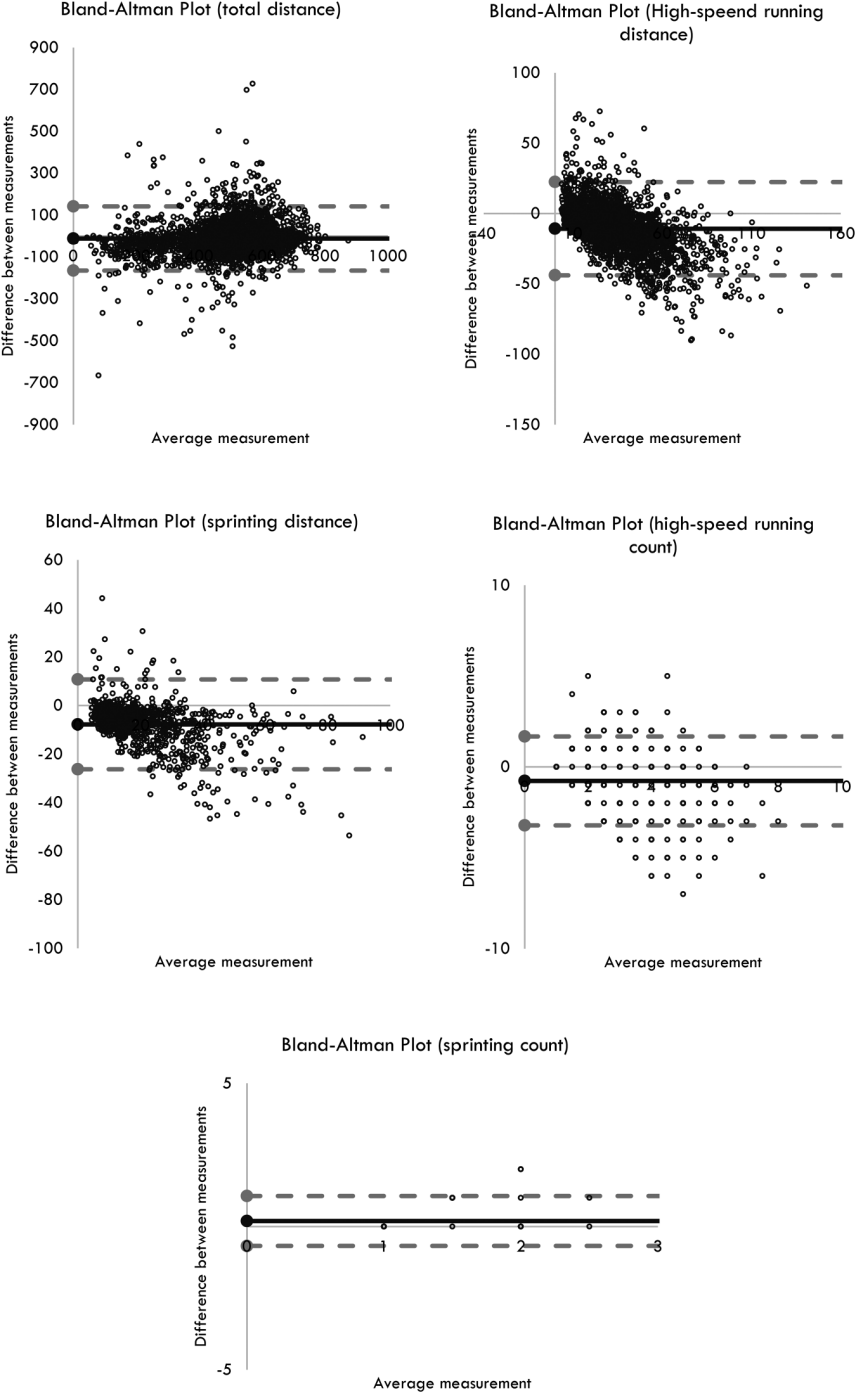
Difference against mean for running-based measures.

The measurement agreement was performed by visual inspection of the data. Figure 2 presents the Bland–Altman plots for the different running-based measurements. The mean difference for TD (for 5-min epochs) was −11.5 [95% CI: −165; 142], while for HSR distance was −11 [95% CI: −44; 22] and for sprinting −8.0 [95% CI: −26; 11]. Regarding HSR and SCs, the mean difference was −1 [95% CI: −3; 2] and 0 [95% CI: −1; 1].

 Table 2 presents descriptive statistics of the running-based measures collected in both the Catapult and Tracab systems. *t*-test revealed significant differences between Catapult and Tracab for TD (*p* < 0.001; *d* = −0.084), HSR distance (*p* < 0.001; *d* = −0.481), sprint distance (*p* < 0.001; *d* = −0.513), HSRC (*p* < 0.001; *d* = −0.558), and SC (*p* < 0.001; *d* = −0.334).

**Table 2.** Descriptive statistics (mean ± standard deviation) for the different running-based measures collected by 5-min epoch and inferential comparisons.

	Catapult	Tracab	Mean difference (Catapult − Tracab)	*p*-value	*d*
Total distance (m) for a 5-min epoch	491.0 ± 116.6	502.7 ± 144.8	−11.8 [95% CI: −14.4; −9.2]	<0.001	−0.084 [95% CI: −0.103; −0.065]
High-speed running (m) for a 5-min epoch	27.5 ± 19.1	38.2 ± 24.0	−10.8 [95% CI: −11.4; −10.1]	<0.001	−0.481 [95% CI: −0.512; −0.450]
Sprint (m) for a 5-min epoch	18.0 ± 13.5	25.7 ± 15.9	−7.7 [95% CI: −8.4; −7.1]	<0.001	−0.513 [95% CI: −0.560; −0.465]
High-speed running (*n*) for a 5-min epoch	2.1 ± 1.2	2.9 ± 1.5	−0.8 [95% CI: −0.8; −0.7]	<0.001	−0.558 [95% CI: −0.597; −0.519]
Sprint (*n*) for a 5-min epoch	1.2 ± 0.4	1.4 ± 0.6	−0.2 [95% CI: −0.2; −0.1]	<0.001	−0.334 [95% CI: −0.408; −0.261]

## Discussion

The objective of this study was to evaluate the absolute agreement of TD, HSR distance, and sprint distance between the 10-Hz Catapult S7 GNSS and the 25-Hz Tracab optical video tracking system. This evaluation was conducted using data collected in 5-min epochs. The main findings showed excellent absolute agreement between the systems for TD and good absolute agreement for HSR distance, sprint distance, HSRC, and SCs. Nonetheless, it was noted that Catapult underestimated the values in comparison to Tracab, particularly for HSR and sprint distances/counts. This corresponds with the findings of previous studies that revealed higher values for tracking systems when compared with GNSS^6^

Regarding TD, the present study showed higher values for Tracab in comparison to Vector S7, which was also previously confirmed.^6^ Moreover, the findings concerning speed values, higher for the tracking system when compared to GNSS, are similar to the findings of previous research.^7^ The observed discrepancies may arise from disparities in data filtering methodologies employed by device software. Notably, the implementation of filters such as moving averages has been shown to yield more refined speed data. However, it is crucial to elucidate that these filtering techniques do not affect the fundamental principle of peak velocity. Peak velocity signifies the utmost speed attained within a specified timeframe, irrespective of any averaged HSR or sprinting encompassed during the said interval. It is noteworthy that the present study adopted 5-min epochs as the temporal units for data analysis.

This study also found that the above differences tend to increase significantly with higher speed distances and counts, which was found in the previous study that analyzed Tracab and GNSS (GPEXE®, Exelio, Udine, Italia) with different epochs (15, 30, and 45 min).^21^ Hence, it can be inferred that as the velocity achieved during high-speed distances increases, the likelihood of encountering greater differences between the systems also amplifies.

In addition, the number of HSR and sprint efforts detected was the greatest for TRACAB, which is also in line with a previous study.^6^ In this regard, it is important to acknowledge that the count detection of speed distances requires a minimum duration above a fixed velocity. For instance, Varley et al.^22^ showed moderate to large differences when different minimum effort durations were applied in the number of HSR detected with 10-Hz GNSS during a soccer match (∼150 efforts detected for 0.1 s duration compared to ∼90 efforts detected for 1 s duration). Thus, when comparing different systems, it is relevant to consider filtering technology differences to understand the pros and cons of each system^23^ as well as other factors, such as the sampling rate, the number of satellites, HDOP, and the software analysis of different systems.^7^

A limitation of the study is a small sample size of participants and, consequently, speculation that a larger sample size could potentially provide a different result when comparing both systems. However, in the context of professional soccer matches, collecting data by means of two different systems is complex and limits the opportunity to gather larger sample sizes. Nonetheless, this had also been pointed as a limitation due to non-ecological environments by the simulation of circuits or matches.^24^ Besides, such devices are expensive and not all teams have access to them. The lack of analysis of accelerometer-based variables is another limitation that may provide further state-of-the-art knowledge, considering that accelerating or decelerating with or without changing direction has been reported as very important in soccer.^25^ Therefore, the investigation with a similar design but with a larger sample size and analysis of accelerometer-based variables is recommended for future studies.

## Conclusions

Although both systems present a strong relationship and acceptable agreement for TD, the interchangeability should be considered cautiously, mainly regarding significant differences in the collected measures. Coaches and sports scientists should be mindful of the differences between both systems when comparing data and avoid using them interchangeably.
